# Breaking barriers in prostate cancer: the mRNA vaccine breakthrough and what comes next

**DOI:** 10.1038/s41541-025-01358-9

**Published:** 2026-01-07

**Authors:** Rayan Rajabi, Somayeh Vafaei, Fatemeh Afrashteh, Roya Ghods, Zahra Madjd

**Affiliations:** 1 Oncopathology Research Center, Iran University of Medical Sciences, Tehran, Iran; 2 School of Medicine, Iran University of Medical Sciences, Tehran, Iran; 3 Department of Molecular Medicine, Faculty of Advanced Technologies in Medicine, Iran University of Medical Sciences, Tehran, Iran

**Keywords:** Cancer, Immunology, Oncology

## Abstract

Prostate cancer (PC) treatment is evolving beyond conventional therapies. This review explores mRNA-based vaccines as a promising immunotherapy. We discuss their mechanism of action, advantages in production and safety, and results from early clinical trials (e.g., CV9103/4). Key challenges like antigen selection and the immunosuppressive tumor microenvironment are addressed, alongside advancements in lipid nanoparticle delivery and combinatorial strategies with checkpoint inhibitors to enhance efficacy and usher in personalized PC treatment.

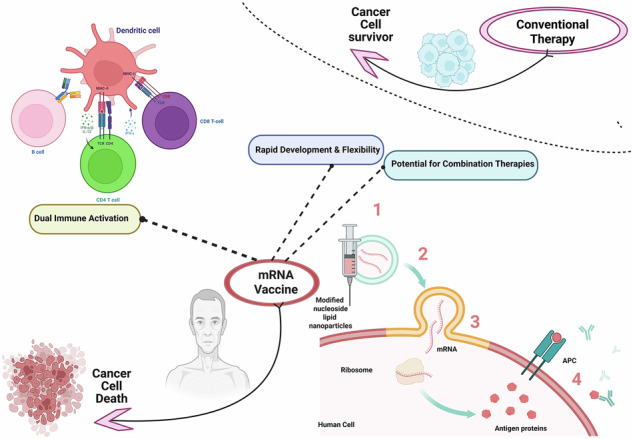

## Introduction

Prostate cancer (PC) continues to be one of the deadliest cancers affecting men globally; PC is both the most prevalent cancer diagnosis and a leading contributor to male cancer deaths^1^. Despite significant advancements in conventional therapies, including surgery, radiotherapy, chemotherapy, hormonal therapy, and targeted therapy, the clinical management of PC continues to face challenges^2,3^. High rates of recurrence, metastatic progression, and the unavoidable onset of therapeutic resistance underscore the limitations of current approaches. These hurdles are particularly evident in metastatic castration-resistant PC (mCRPC), where the disease often exhibits aggressive biological behavior, immune evasion, and a poor prognosis. Thus, there remains an urgent need for novel therapeutic strategies that can effectively target advanced PC while minimizing systemic toxicity^4,5^.

In recent years, messenger RNA (mRNA) vaccines have emerged as groundbreaking immunotherapeutic platforms with transformative potential in oncology. Initially, propelled to global prominence by their success during the COVID-19 pandemic against SARS-CoV-2, mRNA vaccines are now being repurposed to combat cancer, including PC. Unlike traditional therapies that directly attack cancer cells, mRNA vaccines reprogram the host immune system to discriminate and eliminate malignant cells through highly specific antigen-driven responses^6^. This paradigm shift offers several unique advantages such as precision targeting, rapid development, and favorable safety profiles^7,8^.

Nevertheless, the translational pathway from laboratory discovery to clinical application presents important hurdles. The immunosuppressive TME, antigen heterogeneity, and immune escape mechanisms of PC pose significant barriers to the efficacy of vaccines. Early clinical trials, such as those evaluating BioNTech’s CV9104 vaccine and Moderna’s personalized neoantigen vaccine, have demonstrated promising immunogenicity but mixed clinical outcomes, highlighting the need for optimized delivery systems, rational combination strategies, and biomarker-driven patient selection^9^.

Additionally, the integration of mRNA vaccines into the PC treatment represents not only an incremental advance but also a paradigm shift that could redefine therapeutic strategies from early-stage disease to mCRPC. We stand at the intersection of scientific innovation and clinical application^10^.

In this comprehensive review, we survey the evolution of mRNA vaccines for PC, beginning with their molecular design and mechanisms of action. We critically assess preclinical breakthroughs, clinical translation efforts, and ongoing trials that are forming the future of PC immunotherapy. Furthermore, we examine cutting-edge innovations such as artificial intelligence (AI)-driven antigen selection, next-generation lipid nanoparticles (LNPs), and combination therapies, that aim to overcome current limitations. Through integrating molecular biology, immunology, and clinical oncology, this review outlines a framework for further refining mRNA vaccines into precision oncology solutions for PC treatment^11,12^.

## Immunosuppressive mechanisms in PC: key players and pathways

PC employs a multifaceted immunosuppressive network dominated by specific cellular players. Regulatory T cells (Tregs) and myeloid-derived suppressor cells (MDSCs) are the primary orchestrators that actively inhibit cytotoxic T-cell function through two complementary mechanisms. First, they engage immune checkpoint pathways by interacting with cytotoxic T-lymphocyte associated protein 4 (CTLA-4) and programmed cell death protein 1 (PD-1), which are expressed on T cells. These interactions occur via the ligands expressed on Tregs and MDSCs (such as B7 molecules for CTLA-4 and PD-L1/PD-L2 for PD-1), and they deliver inhibitory signals that directly suppress T-cell activation. Second, these immunosuppressive cells secrete inhibitory cytokines, including interleukin-10 (IL-10) and transforming growth factor-beta (TGF-β), which further suppress the proliferation, differentiation, and effector functions of T lymphocytes^13^. Tumor-associated macrophages (M2-TAMs) further reinforce this immunosuppressive state by promoting angiogenesis through vascular endothelial growth factor (VEGF) while secreting anti-inflammatory cytokines. Even cancer-associated fibroblasts (CAFs) contribute by creating physical barriers through extracellular matrix deposition and secreting chemokines such as C-X-C motif chemokine 12 (CXCL12), which exclude effector T cells from the tumor core. These cellular interactions lead to tumor progression and undermines immunotherapy responses^14^. The challenge for mRNA vaccines in PC is less about generating an immune response and more about overcoming the suppressive TME. Even if a vaccine successfully primes cytotoxic T cells, the PC TME is often preventing infiltration or causing functional inactivation. This suppression is driven by MDSCs (via nutrient depletion and ROS), Tregs, M2 macrophages (secreting IL-10, TGF-β), and a metabolically hostile niche that induces T-cell exhaustion. The precedent set by Sipuleucel-T is clear: immune activation in PC is possible, but clinical efficacy is limited by the TME’s suppression of those activated cells. Since a high burden of these same immunosuppressive cells predicts failure for other immunotherapies, it is a well-justified expectation that mRNA vaccine efficacy would be similarly constrained^15^.

At the molecular level, PCs exploit ICIs and metabolic sabotage to evade immunity. The PD-1/PD-L1 axis, although less prominent in PC than in other cancers, remains a key resistance mechanism, whereas CTLA-4 overexpression on Tregs further dampens T-cell activation. Soluble factors such as TGF-β and IDO (indoleamine 2,3-dioxygenase) starve T cells, which are critical resources, and TGF-β drives Treg differentiation, whereas IDO depletes tryptophan, triggering T-cell anergy^16^. Hypoxia, a hallmark of advanced PC, amplifies immunosuppression by upregulating hypoxia-inducible factor 1-alpha (HIF-1α)-dependent genes such as CD47 (a “don’t eat me” signal) and VEGF, which simultaneously blocks immune infiltration and fuels angiogenesis. These pathways collectively establish a molecular shield against immune attack^17^.

The TME of PCs integrates these cellular and molecular mechanisms into a spatially organized immunosuppressive ecosystem. Hypoxic regions recruit MDSCs and M2-TAMs, while dense fibrotic stroma from CAFs physically impedes T-cell trafficking. Exosomes secreted by PC cells circulate immunosuppressive cargos (e.g., PD-L1 and TGF-β) to distant lymphoid tissues, systemically inhibiting immunity^18,19^. Androgen receptor (AR) signaling adds another layer by upregulating IL-23 to expand Tregs and increase PD-L1 expression. This dynamic, spatially coordinated immunosuppression explains why monotherapies often fail in PC and underscores the need for combinatorial strategies targeting both tumor cells and their protective niches^20^.

**Fig. 1:**
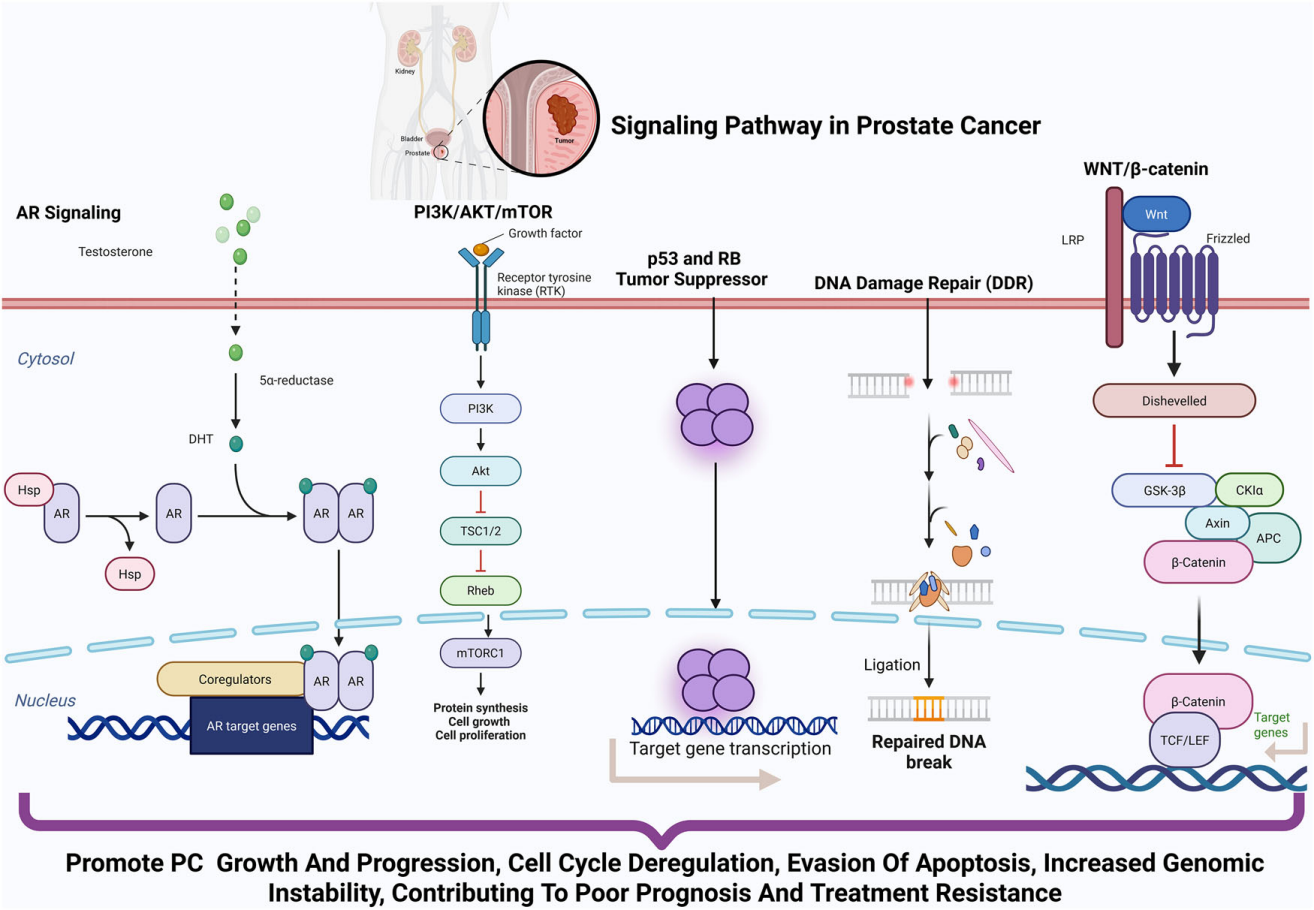
Key signaling pathways and molecular alterations driving PC progression and therapeutic resistance. This schematic illustrates the major molecular pathways implicated in PC development, progression, and therapeutic resistance, highlighting key molecules, their functional roles, and therapeutic targets: Androgen Receptor (AR) Signaling: The central pathway in PC pathogenesis. AR, upon binding to androgens, translocates to the nucleus and activates transcription of genes promoting cell proliferation and survival. Despite androgen deprivation therapy (ADT), AR signaling persists in castration-resistant prostate cancer (CRPC) via AR gene amplification, point mutations, splice variants (e.g., AR-V7), and intratumoral androgen biosynthesis. AR remains the principal therapeutic target in both hormone-sensitive and CRPC stages. PI3K/AKT/mTOR pathway: frequently activated due to PTEN loss (in 40–60% of cases), this pathway enhances tumor cell survival, proliferation, and metabolism. PTEN deficiency leads to unchecked PI3K/AKT signaling and contributes to therapy resistance. Key therapeutic targets include PI3K, AKT, and mTOR inhibitors, especially in PTEN-deficient tumors. p53 and RB tumor suppressor pathways: inactivating mutations in TP53 (observed in ~50% of metastatic PC) and RB1 loss are associated with high-grade, aggressive disease. These disruptions lead to cell cycle deregulation, evasion of apoptosis, and increased genomic instability, contributing to poor prognosis and resistance to conventional therapies. DNA Damage Repair (DDR) Pathways: Defects in homologous recombination repair (HRR) are observed in 20–25% of metastatic PC cases. Key genes involved include BRCA1, BRCA2, and ATM, whose loss renders tumors sensitive to PARP inhibitors (e.g., olaparib) and platinum-based chemotherapy, offering a precision medicine approach. WNT/β-catenin signaling: aberrant activation of this pathway via mutations or epigenetic alterations promotes tumor cell proliferation, EMT, and maintenance of CSCs. Targeting WNT ligands or β-catenin signaling components is under investigation as a therapeutic strategy.

**Fig. 2:**
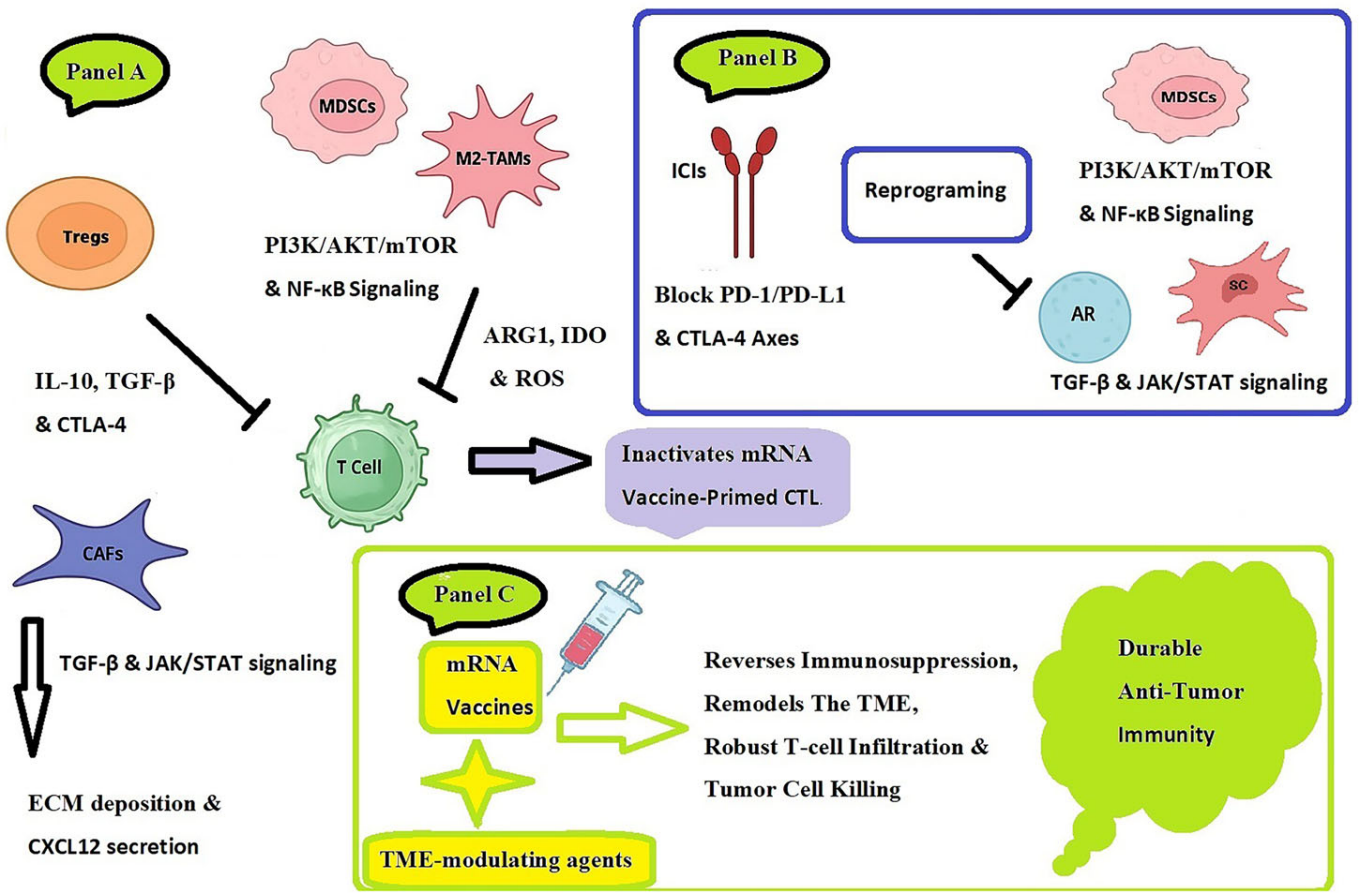
The immunosuppressive network in prostate cancer and rational combinatorial strategies to enable mRNA vaccine efficacy. (Panel A) The PC TME is characterized by a multifaceted immunosuppressive network. Myeloid-derived suppressor cells (MDSCs) and tumor-associated macrophages (M2-TAMs), driven by PI3K/AKT/mTOR and NF-κB signaling, suppress T cells via metabolic enzymes (ARG1, IDO) and ROS. Regulatory T cells (Tregs) inhibit T-cell function through cytokine secretion (IL-10, TGF-β) and immune checkpoint engagement (CTLA-4). Cancer-associated fibroblasts (CAFs) create a physical barrier via ECM deposition and CXCL12 secretion, promoted by TGF-β and JAK/STAT signaling. Collectively, this inactivates mRNA vaccine-primed cytotoxic T cells. (Panel B) Targeted therapeutic interventions are designed to disrupt this suppressive network. These include: immune checkpoint inhibitors (ICIs) to block PD-1/PD-L1 and CTLA-4 axes; pathway inhibitors to reprogram myeloid cells (PI3K/AKT/mTOR, NF-κB) and stromal cells (TGF-β, JAK/STAT); stromal disruptors to break down physical barriers; and androgen receptor blockers. (Panel C) The integration of mRNA vaccines with these TME-modulating agents reverses immunosuppression, remodels the TME, and allows for robust T-cell infiltration and tumor cell killing, leading to durable anti-tumor immunity.

For the development of effective therapeutic strategies in PC, a major challenge is overcoming the molecular pathways that drive disease progression and therapeutic resistance. Key intrinsic pathways, as illustrated in Fig. 1, represent promising targets for intervention. These include the persistent Androgen Receptor (AR) Signaling in mCRPC, the frequently activated PI3K/AKT/mTOR pathway often due to PTEN loss, and the inactivated p53 and RB tumor suppressor pathways. Furthermore, targeting vulnerabilities such as defects in DNA Damage Repair (DDR) Pathways (e.g., in BRCA1/2) with Poly (ADP-ribose) polymerases (PARPs) inhibitors, and investigating strategies against aberrant WNT/β-catenin signaling, are critical for developing more effective and personalized treatments^21,22^. The integration of mRNA vaccines with targeted TME-modulating agents such as ICIs, stromal-targeting therapies (e.g., FAP inhibitors such as sibrotuzumab, CXCL12/CXCR4 antagonists such as plerixafor, or TGF-β pathway inhibitors such as galunisertib), or androgen signaling blockers may provide a path to overcome the immune evasion mechanisms of PC and achieve durable antitumor immunity^23,24^,

The central premise is that these pathways are key hubs in the immunosuppressive TME, and their targeted inhibition can shift the balance to favor vaccine efficacy. For instance, inhibiting the PI3K/AKT/mTOR pathway can suppress tumor growth while paradoxically fostering durable memory T-cells. Similarly, blocking the TGF-β pathway can prevent the functional silencing of vaccine-primed T-cells, reduce regulatory T-cell activity, and remodel the fibrotic stroma to improve T-cell infiltration. (Fig. 2)

The therapeutic strategy is nuanced and pathway-specific. For the JAK/STAT pathway, the goal is context-dependent inhibition blocking nodes activated by immunosuppressive cytokines like IL-6 while preserving its function in T-cells for robust activation. The NF-κB pathway presents a dual opportunity: inhibiting its constitutive signaling in tumor cells to reduce survival and immunosuppression, while potentially enhancing its activity in antigen-presenting cells (APCs) to improve the initial priming of the vaccine response. By detailing these mechanisms, we more clearly articulate the rationale for how modulating these pathways can therapeutically enhance mRNA vaccine-induced anti-tumor immunity^25,26^.

## Cancer immunotherapy in PC: current paradigms and emerging strategies

The landscape of PC therapy has been transformed by recent breakthroughs in cancer immunotherapy^27–29^. By utilizing the patient’s immune response to identify and destroy tumor cells precisely, such methods avoid the shortcomings of conventional therapies including non-specific cytotoxicity to healthy tissues, dose-limiting systemic toxicities, incomplete eradication of minimal residual disease, rapid emergence of drug resistance, and the absence of durable immune memory that contributes to high relapse rates^30–32^. Within this paradigm, cancer vaccines have emerged as a leading strategy, uniquely designed to activate rather than supplant innate immunity. Unlike chemotherapy or radiotherapy, which directly kill cancer cells, vaccines elicit durable, antigen-specific responses by educating the immune system to recognize tumor-associated antigens (TAAs) or tumor-specific antigens (TSAs)^32^.

## Monoimmunotherapy in PC

For PC vaccines to be effective, they must successfully transport tumor-associated/specific antigens (TAAs/TSAs) to APCs, triggering sequential immune reactions that stimulate both cytotoxic CD8⁺ T cells and CD4⁺ T cells, which can also exert direct tumor-killing functions. These coordinated responses are essential for generating durable immunological memory^33,34^. This phenomenon is especially important in PC, a malignancy attributed to significant inter- and intratumoral antigenic heterogeneity. Unlike highly immunogenic malignancies such as melanoma, PC is characterized by an immunologically quiescent TME with a low neoantigen burden and weak baseline immune infiltration. These features pose challenges for vaccine efficacy, necessitating platforms capable of delivering patient-specific neoantigens and overcoming the immunosuppressive TME^35,36^. Despite its FDA approval, Sipuleucel-T provides only modest clinical benefit. This autologous cellular immunotherapy is manufactured by collecting a patient’s peripheral blood mononuclear cells (PBMCs), exposing them ex vivo to a recombinant fusion protein (PA2024) composed of prostatic acid phosphatase (PAP) linked to granulocyte–macrophage colony-stimulating factor (GM-CSF), and then reinfusing the activated PBMCs back into the patient. In the pivotal IMPACT phase III trial, sipuleucel-T improved median overall survival (OS) by ~4.1 months (25.8 vs. 21.7 months with placebo) but did not significantly delay objective disease progression, and immune activation was largely limited to antigen-specific T-cell responses without robust tumor regression in most patients. These modest effects underscore the need for next-generation vaccine strategies, such as mRNA platforms or dendritic-cell-based vaccines tailored to individual tumor profiles, to increase antigen presentation and cytotoxic T lymphocyte (CTL) priming^37^.

Checkpoint inhibitors (ICIs), specifically those targeting PD-1, PD-L1, and CTLA-4, have revolutionized treatment in several malignancies but have demonstrated limited efficacy as monotherapies in PC. The immunologically inert TME, which is characterized by poor T-cell infiltration, a low mutational burden, and the presence of suppressive cytokines (e.g., IL-10 and TGF-β), dampens the potential of ICIs^12,38^.

As a result, clinical trials in mCRPC patients have shown minimal response rates when ICIs are used alone. Similarly, cytokine-based therapies—such as IL-2 or IFN-α, which are theoretically capable of enhancing immune activation—have been limited by systemic toxicity and nonspecific immune stimulation^39,40^. Targeted monoclonal antibodies such as ^177Lu-PSMA-617 (radiolabeled anti- prostate-specific membrane antigen (PSMA)) provide a passive form of immunotherapy that delivers tumor-directed cytotoxicity but does not induce lasting immunologic memory. Similarly, small-molecule inhibitors that target immunosuppressive pathways such as TGF-β, IDO, or A2AR aim to modulate the TME but require precise patient stratification and rational combination strategies to achieve clinical benefit. For ICIs, patients with microsatellite instability-high (MSI-High) or mismatch repair-deficient (dMMR) tumors, as well as those with high CD8⁺ T-cell infiltration, are more likely to respond. Stratifying patients based on these biomarkers can optimize therapeutic outcomes and guide the selection of combination regimens^41^.

CAR-T-cell therapy represents an active, cell-based immunotherapy that has proven successful in treating hematologic malignancies, but this approach remains in the experimental stage in solid tumors such as PC. The primary challenges stem from the hostile prostate TME, which acts as both a physical barrier and an immunological barrier^42^. Dense stromal tissue impedes T-cell infiltration, whereas a cocktail of immunosuppressive factors such as TGF-β, adenosine, and MDSCs inhibits CAR-T-cell function. Furthermore, antigen heterogeneity and downregulation, particularly of PSMA, compromise antigen recognition, with loss rates approaching 38% by 12 weeks post treatment^43^.

These obstacles are compounded by manufacturing challenges, including the need for a balanced CD4+/CD8+ ratio and enrichment for Central Memory T (TCM) cells, which demonstrate greater persistence but are scarce in heavily pretreated patients. Although preclinical and early-phase clinical studies have shown some promise, the multifaceted resistance mechanisms in PC limit the durability and scalability of CAR-T-cell monotherapy, necessitating combination strategies to fully unleash its therapeutic potential^42,44^.

All cancers, including PC, continue to present a clinical challenge due to their recurrence and progression, which are often governed by a subpopulation of cancer stem cells (CSCs). These cells possess self-renewal capacity, tumor-initiating potential, and resistance to standard treatments^45^. CSCs evade therapy through mechanisms such as cellular quiescence, enhanced DNA repair, and the expression of drug efflux transporters, contributing to therapeutic resistance and metastatic spread, particularly to bone, a common site in advanced PC. Their dynamic interaction with the TME further facilitates immune evasion and metastasis, underscoring their role as critical therapeutic targets^46^. These cells often lack or express low levels of AR and prostate-specific antigen (PSA) and may not express PAP, making them less susceptible to therapies such as Sipuleucel-T. Recent studies suggest that targeting PCSC-associated markers such as CD44 and EpCAM through novel immunotherapies could overcome this limitation and improve patient outcomes.

Tumor heterogeneity, immunosuppressive signaling pathways, and the absence of well-defined TSAs limit the effectiveness of current vaccine approaches. Currently, some vaccine strategies focus on eliminating CSCs by incorporating CSC-specific antigens such as CD44, ALDH1, and CD133 into mRNA vaccine platforms or Dendritic Cell (DC)-based vaccines^47^. Preclinical data support the efficacy of these approaches, showing reduced tumorigenicity and enhanced antitumor immunity^12^.

**Scheme 1:**
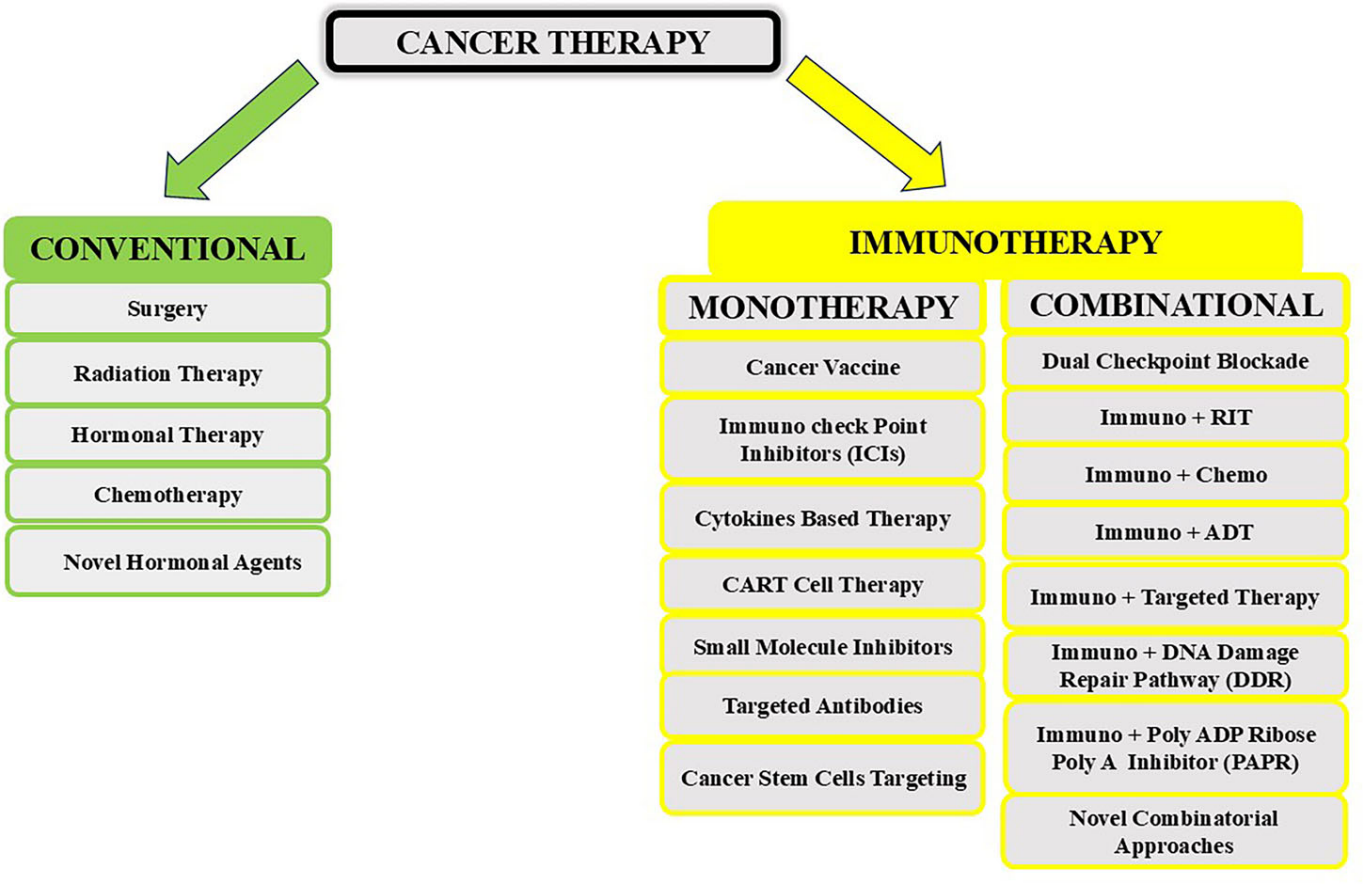
Conventional and Immuno (mono and combinational) therapy are considered as cancer treatment.

Future therapeutic strategies are increasingly focused on integrating CSC-targeted vaccines with other modalities to overcome resistance. Combining vaccines with signaling pathway inhibitors (e.g., Hedgehog, Wnt, and Notch), epigenetic drugs (e.g., HDAC inhibitors), or radiotherapy may increase antigen presentation and CSC immunogenicity^48^. Concurrent use of ICIs such as PD-1 or CTLA-4 blockade can also help overcome immune evasion. However, critical challenges remain, including the identification of reliable CSC biomarkers, managing off-target effects (especially in CAR-T-cell therapy), and minimizing toxicity to normal stem cells. Continued refinement of these approaches, guided by an improved understanding of CSC biology, holds promise for achieving more durable responses in advanced PC^49,50^, which are summarized in Scheme 1.

## Combination immunotherapy in PC

PC presents unique challenges for effective immunotherapy, largely due to its immunosuppressive TME, low mutational burden, and heterogeneous antigen expression. Given the limited efficacy of monotherapies in this context, recent advances have focused on combination immunotherapy strategies that aim to overcome immune resistance and enhance antitumor responses^51^. These approaches work by leveraging complementary mechanisms of action, such as remodeling the TME, promoting antigen presentation, and activating cytotoxic immune responses. Key strategies include combining ICIs with traditional therapies such as ADR, radiotherapy, chemotherapy, PARP inhibitors, and targeted treatments which summarized in Table 1. Early-phase clinical trials have produced encouraging reports, particularly in molecularly selected patients, highlighting the potential of rational combinatorial regimens to increase the therapeutic potential of immune-based treatments in PC^52^.

**Table 1:** Current and emerging prostate cancer therapies and opportunities for integration with mRNA vaccines

Therapy	Mechanism of action	Key limitations	Immunologic effect	Opportunities for mRNA vaccine integration
Androgen Deprivation Therapy (ADT)	Inhibits androgen receptor signaling, leading to tumor regression and suppression of tumor growth.	Most patients eventually develop castration-resistant disease; ADT is not curative in advanced stages.	ADT can increase immune cell infiltration and modulate the tumor microenvironment.	Combining ADT with mRNA vaccines may enhance immune activation and improve vaccine efficacy.
Radiotherapy	Induces DNA damage, resulting in tumor cell death and potential release of tumor antigens.	Primarily local effects; limited systemic control; risk of relapse and progression in advanced disease.	Promotes antigen release, enhances immune visibility of tumors, and can facilitate immune priming.	Radiotherapy can be used to increase tumor immunogenicity and improve vaccine-induced immune responses.
Chemotherapy	Induces direct cytotoxicity against proliferating tumor cells.	Associated with systemic toxicity, immunosuppression, and resistance development.	Can induce immunogenic cell death, leading to the release of tumor-associated antigens.	Chemotherapy can enhance antigen availability, providing a more favorable environment for mRNA vaccine activity.
PARP inhibitors	Target DNA damage repair pathways, leading to synthetic lethality in tumors with DDR deficiencies.	Benefit is mainly restricted to patients with homologous recombination repair gene mutations; resistance may occur.	Can increase genomic instability and neoantigen generation.	PARP inhibition may synergize with mRNA vaccines by increasing tumor neoantigen load and immune recognition.
Immune Checkpoint Inhibitors (ICIs)	Block inhibitory pathways that suppress T-cell activity, promoting immune-mediated tumor killing.	Response rates remain low in unselected PC populations due to low tumor mutational burden and immunosuppressive TME.	Reinvigorate exhausted T cells and enhance antitumor immune responses.	mRNA vaccines can provide neoantigen-specific T cell priming, potentially increasing the proportion of patients who benefit.

Although over the past decades, treatment options for PC have improved patient outcomes, they remain limited by resistance, toxicity, and lack of durable disease control in advanced stages. Resistance to standard therapies, immune exclusion, and tumor heterogeneity are major barriers to long-term benefit. mRNA vaccines offer unique opportunities to address these challenges by enhancing tumor immunogenicity, priming patient-specific immune responses, and synergizing with existing treatment modalities. This integration has the potential to reshape the therapeutic landscape, shifting the focus from incremental survival benefits to durable immune-mediated control^53^.

Emerging evidence also supports the potential of combining local ablative techniques such as radiofrequency ablation (RFA) with immunotherapy. RFA can induce immunogenic cell death and release TAAs, thereby promoting DC activation and systemic immune priming. When followed by immunotherapies such as neoantigen vaccines, this approach may amplify antigen-specific T-cell responses and improve systemic tumor control. Although most clinical data originate from pan-cancer studies, early findings suggest that integrating RFA with immunotherapy could be a feasible and synergistic approach for PC, warranting further investigation in disease-specific trials^54^.

However, combining ICIs with other treatments offers promising potential to overcome immune suppression and improve therapeutic responses: ICI+ADT has been shown to increase the infiltration of tumor-infiltrating lymphocytes (TILs), which could increase the efficacy of PD-1 blockade by promoting immune activation within the TME^55^. ICI + Radiotherapy: induce cellular apoptosis and subsequent release of tumor antigens that can stimulate T-cell responses. The combination of radiotherapy with ICIs has the potential to increase immune recognition by tumor cells and further activate antitumor immunity. ICI + Cancer Vaccines: Combining ICIs with cancer vaccines, such as mRNA vaccines, can prime T cells while preventing their exhaustion, creating a synergistic effect that enhances immune responses (e.g., BioNTech trials combining CV9104 with avelumab)^56^.

Radioimmunotherapy (RIT) combines radiotherapy with immunotherapy, representing a novel approach for treating PC. In this strategy, a radionuclide is conjugated to a monoclonal antibody (mAb) that targets specific tumor antigens, such as PSMA, STEAP1, or prostate stem cell antigen (PSCA). The antibody directs radiotherapy specifically to tumor cells, enhancing the accuracy of radiotherapy targeting while preserving healthy tissues/organs^57^.

When chemotherapy combined with ICIs, can help reverse the immunosuppressive TME, improving immune cell infiltration and activation. This approach aims to create a more favorable environment for immune responses while also directly targeting tumor cells^58^.

The mainstay therapy for advanced PC is ablation of androgen signaling to inhibit tumor progression. Surprisingly, it also remodels the immune landscape by augmenting CD8+TIL infiltration and function and sensitizing PCs to ICIs. The combination of ICIs with ADR can enhance antitumor immunity by increasing the number of immune cells within the TME and overcoming immune suppression^59^. and synergistically address both the molecular drivers of tumors and their ability to evade immune detection^60^.

Tumors with defects in DDR pathways, such as those harboring mutations in genes such as BRCA1/2, are often more sensitive to treatments that induce DNA damage. Combining immunotherapy with DDR inhibitors, such as PARP inhibitors, has drawn considerable interest as a potential strategy for treating PC. DDR inhibition can increase tumor cell vulnerability to DNA-damaging agents and enhance the display of tumor antigens, thereby promoting more robust immune detection and response. This combination could lead to more effective antitumor immunity, particularly in tumors with defective DDR mechanisms^61^.

PARP inhibitors, which target tumors with defective DNA repair mechanisms, have gained prominence as integral tools in oncologic care. In PC, particularly in tumors with BRCA mutations, PARP inhibitors can enhance the effects of immunotherapy. Through the induction of DNA damage and subsequent tumor antigen release, PARP inhibitors can potentiate antitumor immunity. Combination with ICIs, this approach aims to increase tumor immunogenicity, leading to enhanced activation of T cells and destruction of cancer cells^62^.

**Scheme 2:**
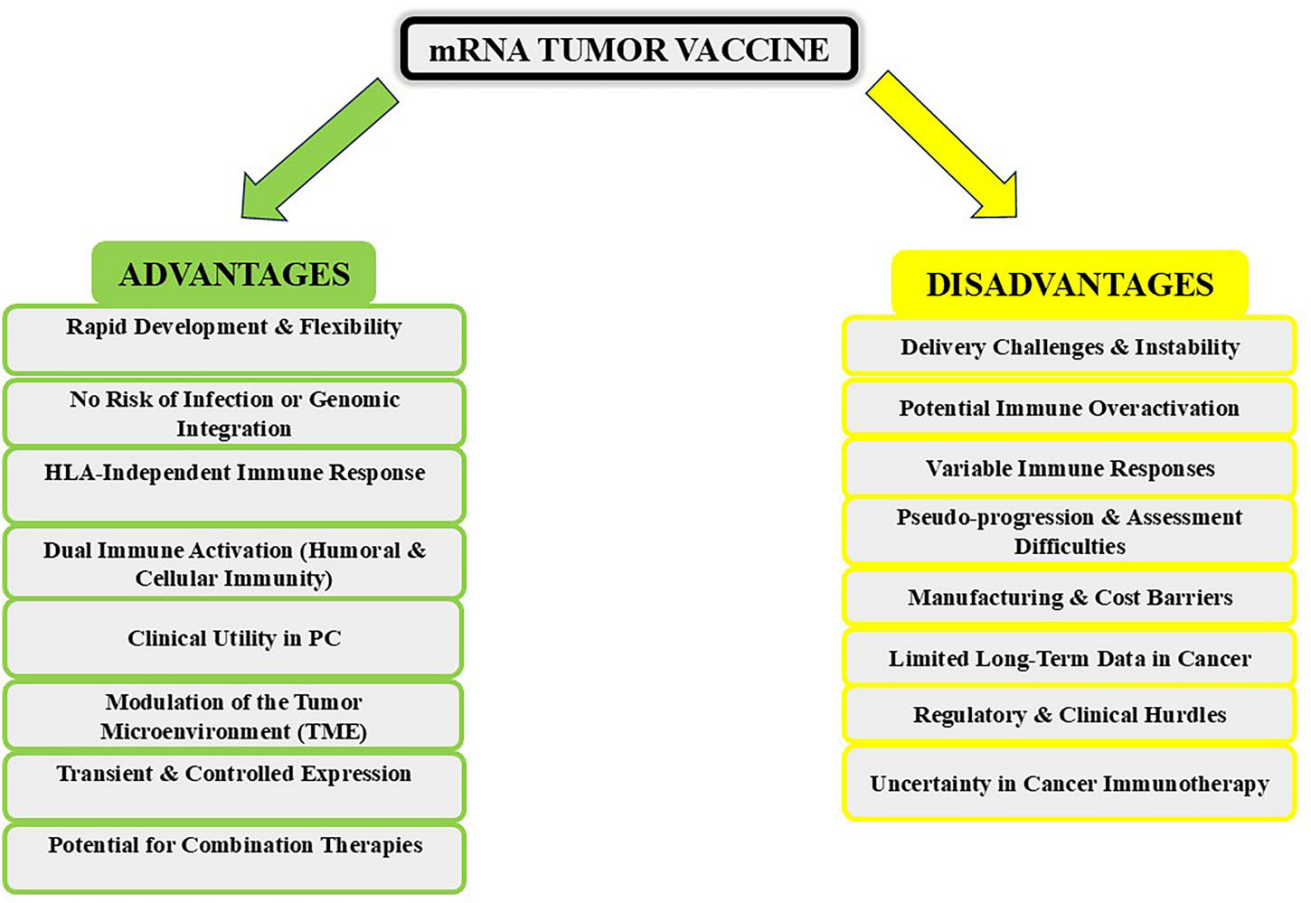
The list of the mRNA vaccine advantages and disadvantages.

In addition to established combination strategies, several novel approaches are being explored to enhance immunotherapy efficacy in PC (summarized in Scheme 2). Oncolytic viruses represent one such strategy; these engineered viruses selectively infect and lyse tumor cells while simultaneously stimulating the immune system by releasing tumor antigens and promoting dendritic cell activation within the TME^63^. Bispecific antibodies offer another promising modality by simultaneously targeting two distinct antigens, enabling precise tumor recognition while activating immune effector cells, including T cells, to directly eliminate cancer cells. Metabolic modulation is also gaining attention, as tumor cells often rely on altered metabolic pathways that support growth and immune evasion. Therapeutic interventions targeting these pathways can reprogram the TME, improving immune cell function and enhancing the efficacy of immunotherapies. Finally, microbiome modulation has emerged as a complementary strategy, as the intestinal microbiota profoundly influences systemic and local immune responses. Modifying the microbiome has been shown to increase immune activation and enhance responses to immunotherapies, highlighting its potential role in improving treatment outcomes for PC^64^.

In summary, combination immunotherapy offers a promising multipronged strategy to overcome the immunosuppressive and heterogeneous nature of PC. Approaches such as dual immune checkpoint blockade, oncolytic viruses, bispecific antibodies, and other emerging modalities aim to increase immune activation, improve tumor recognition, and counteract resistance mechanisms inherent in the complex biology of PC. Early clinical trials have yielded encouraging outcomes, suggesting that these integrated strategies may significantly improve therapeutic efficacy and potentially transform the treatment landscape for PC in the near future.

## Tumor vaccine toolkit: engineering precision weapons

### Historical foundations: the birth of the tumor vaccine

The foundational concept of tumor vaccines dates back to the groundbreaking work of William B. Coley, who reported remarkable tumor shrinkage in patients diagnosed with bone and soft tissue sarcomas after the administration of ‘Coley Toxins’, a preparation derived from *Streptococcal bacteria*. This landmark discovery paved the way for cancer immunotherapy, emphasizing the potential of leveraging the immune system to fight cancer^35^.

### Tumor vaccine classifications: preventive vs. therapeutic

Tumor vaccines represent a type of active immunotherapy that aims to engage the immune system, to recognize and destroy cancer cells. The primary goals of tumor vaccines are to inhibit tumor growth, prevent recurrence or metastasis, eradicate cancer cells, and prolong patient survival^65^.

Tumor vaccine strategies fall into two primary categories: preventive (prophylactic) and therapeutic. To date, the U.S. FDA has approved only two preventive vaccines, both aimed at cancers caused by hepatitis B virus and human papillomavirus viral infections. At present, no preventive vaccines have been approved for nonviral cancers in humans^66,67^.

Therapeutic tumor vaccines encompass a range of platforms, each characterized by distinct mechanisms of action and immunological profiles, as summarized in Table 2^68^. Peptide-based vaccines are highly specific and cost-effective, targeting well-defined tumor antigens; however, their efficacy can be constrained by low immunogenicity and HLA restriction, limiting applicability to selected patient populations^69^. Dendritic cell (DC) vaccines, in contrast, provide a personalized immunotherapeutic approach by efficiently presenting a diverse repertoire of tumor antigens to T cells and inducing robust immune activation. Nonetheless, their widespread clinical application is hampered by high production costs, labor-intensive preparation, and complex manufacturing processes^70^. Whole-tumor cell vaccines present the advantage of delivering a comprehensive antigenic landscape, thereby minimizing immune escape due to antigen loss. However, these approaches carry the risk of including inhibitory antigens or epitopes within the vaccine formulation, which may attenuate the desired immune response. Additionally, their non-specific nature can lead to off-target effects and challenges with standardization across clinical settings^71^.

**Table 2:** Types of therapeutic tumor vaccines

Vaccine type	Mechanism/Approach	Advantages	Disadvantages	Examples/Details
Peptide-based vaccines	Use short, tumor-associated peptide fragments to stimulate a specific T-cell response.	High safety profile (no live components). Easy and cost-effective to synthesize. Stable and well-defined structure. Can elicit antigen-specific T cell responses. Flexible design—can be customized for specific tumor antigens.	Often weak immunogenicity—requires adjuvants or delivery systems. HLA-restriction limits patient coverage. Risk of immune tolerance or rapid degradation in vivo. Limited induction of CD4⁺ T helper responses if not properly designed.	Target antigens such as HER2/neu, MUC1, or melanoma-associated peptides.
Dendritic cell vaccines	Involve harvesting a patient’s dendritic cells, loading them with tumor antigens ex vivo, and reinfusing them to trigger an immune response.	Highly potent antigen-presenting cells—strong induction of T cell immunity. Personalized—can be loaded with patient-specific tumor antigens. Capable of inducing both CD4⁺ and CD8⁺ T cell responses. Low risk of severe adverse events.	Labor-intensive and expensive to manufacture. Requires ex vivo cell manipulation and specialized facilities. Variable efficacy among patients. Time-consuming preparation, which can delay treatment.	Proven example: Sipuleucel-T for PC.
Whole tumor cell vaccines	Utilize entire tumor cells (either autologous or allogeneic) to provide a broad array of antigens to the immune system.	Broad antigen spectrum—targets multiple tumor antigens simultaneously. No need to identify specific tumor antigens in advance. Can induce both humoral and cellular immunity. Potentially effective against tumor heterogeneity.	Risk of inducing autoimmunity due to shared self-antigens. Variable immunogenicity between patients. Difficult to standardize and manufacture. May require additional immune stimulation (e.g., adjuvants).	Often combined with adjuvants to enhance immunogenicity.
DNA vaccines	Deliver plasmid DNA encoding tumor antigens, which is taken up by host cells that then produce the antigen and initiate an immune response.	Simple, stable, and inexpensive to produce. Long-lasting antigen expression. Can induce both cellular and humoral immune responses. Easy to modify or combine with other immunotherapies.	Generally low immunogenicity in humans. Risk of genomic integration (although very low). Requires delivery enhancement (e.g., electroporation). Regulatory and safety concerns.	Being explored for various solid tumors.
RNA vaccines	Use messenger RNA encoding tumor antigens; once inside cells, the mRNA is translated into protein antigens to activate the immune system.	No risk of genomic integration. Strong and transient antigen expression. Rapid and scalable manufacturing. High flexibility for encoding multiple antigens. Potent induction of innate and adaptive immunity.	RNA instability requires special formulation (e.g., lipid nanoparticles). Cold chain storage and transport are challenging. Potential for strong inflammatory reactions. Short duration of expression may require boosting.	Emerging approach with potential for rapid development.
Viral vector-based vaccines	Employ modified viruses to deliver genes encoding tumor antigens, thus promoting a strong immune response.	High immunogenicity—strong T cell and antibody responses. Efficient antigen delivery and expression in vivo. Can mimic natural infection and induce long-lasting immunity. Versatile platform for different tumor antigens.	Pre-existing immunity to vector can reduce efficacy. Complex manufacturing and regulatory requirements. Risk of anti-vector immune responses limiting repeated dosing. Safety concerns in immunocompromised patients.	Examples include vaccines using adenovirus or poxvirus vectors.
Neoantigen vaccines	Personalized vaccines designed to target patient-specific tumor mutations (neoantigens) that are not present in normal tissues.	Personalized—targets tumor-specific mutations with minimal risk of autoimmunity. Highly specific T cell activation. Can overcome tumor immune evasion. Promising clinical outcomes in early trials.	Requires individualized neoantigen identification (time-consuming and costly). Tumor evolution may lead to loss of targeted neoantigens. Technically complex manufacturing. Efficacy depends on patient’s immune competence.	Custom-tailored using genomic sequencing of individual tumors.

In addition to these strategies, nucleic acid-based vaccines, such as DNA and RNA vaccines, have emerged as innovative options in tumor vaccine development. DNA vaccines offer stability and the ability to encode multiple antigens; however, their clinical effectiveness is frequently limited by low immunogenicity and challenges in efficient delivery. In contrast, RNA vaccines are capable of inducing strong immune responses and can be rapidly produced, but specialized formulations are needed to prevent degradation^72,73^. Viral vector-based vaccines are recognized for their efficient gene delivery and strong capacity to stimulate robust cellular immune responses, but preexisting immunity to the vectors and safety concerns may limit their clinical application.

Finally, neoantigen vaccines take a highly personalized approach by targeting unique mutations within individual tumors^74^. This specificity reduces the risk of damaging normal tissues but comes with challenges such as high costs and time-consuming production owing to the need for individualized genomic sequencing. Overall, Table 2 illustrates the dynamic landscape of tumor vaccine development, where each approach presents trade-offs in terms of specificity, efficacy, cost, advantages, disadvantages, and logistical challenges that must be carefully balanced in the pursuit of effective cancer immunotherapies^75,76^. Each vaccine type is engineered to activate the immune system through distinct mechanisms, offering a variety of strategies to target cancer cells^77^. Like PC, glioblastoma is a highly challenging malignancy, with conventional therapies often failing due to tumor heterogeneity and treatment resistance^78^. As with PCs, key considerations for glioma vaccines include optimal antigen selection and overcoming the immunosuppressive TME, as outlined in Table 3. The experiences with mRNA vaccines in both malignancies underscore their potential as versatile immunotherapy approaches, although further clinical validation is necessary to fully establish their therapeutic role^79^.

**Table 3:** List of antigens used in tumor vaccines

Antigen	Function	Clinical relevance	References
Tumor-associated antigens (TAAs)			
PSMA	Transmembrane glycoprotein	Target for mAbs, CAR-T, and vaccines	^137^
PSCA	Cell signaling	Overexpressed in metastases	^138^
STEAP1	Iron reductase	Associated with poor prognosis	^139^
PAP	Non-specific phosphomonoesterase synthesized in prostate epithelial cells	Increases with prostate cancer progression	^140,141^
PSA	Tumour growth,	Associated with metastatic prostate cancer	^206^
c-MYC	Cancer growth and invasion in vitro and in vivo	Its upregulation is associated with reduced overall survival, clinical stage, lymph node metastasis and undesirable prognosis of PC patients.	^207^
p53	A transcription factor essential for the prevention of cancer formation	Malignant progression and associated with metastatic	^208,209^
MUC-1	Expressed on the apical surface of glandular epithelium protection, adhesion and signaling	Prognostic marker	^210^
PTEN	Negative regulator of the PIK3/Akt survival pathway	The most frequently deleted tumor suppressor gene in prostate cancer	^211^
Tumor-specific neoantigens (TSAs)			
SPOP	Specific targeting of proteins for ubiquitination and subsequent proteasomal degradation	Prognostic and predictive	^212^
FOXA1 mutations	Driver mutations in PC	Drive aggressive PC with lineage plasticity and resistance to AR-targeted therapies.	
TMPRSS2-ERG fusion	Common fusion protein	Defines a molecular subtype with altered DNA repair pathways and potential PARP inhibitor sensitivity.	
Cancer stem cells (CSCs)			
CD44	Cell adhesion, hyaluronan binding	Associated with aggressive PC and castration resistance	^213^
CD133 (Prominin-1)	Stem cell maintenance	Linked to tumor initiation and metastasis	^213^
ALDH1	Detoxification, therapy resistance	Correlates with poor prognosis	^48^
Integrin α2β1	Extracellular matrix interaction	Enriches for tumor-initiating cells	

## Precision targeting: mRNA vaccines in PC

### Revolution of the mRNA vaccine: from COVID-19 to PC

The oncologic application of mRNA vaccines began with DC-based delivery in 2002, when a proof-of-concept trial targeted carcinoembryonic antigen in adenocarcinoma. This landmark study revealed that DCs loaded with tumor antigen-encoding mRNAs were capable of inducing antigen-specific T-cell responses, laying the groundwork for modern mRNA vaccine design^80^. The interest in mRNA vaccines has increased due to the successful deployment of SARS-CoV-2 mRNA vaccines during the COVID-19 pandemic^81,82^. The COVID-19 pandemic has created an urgent demand for the swift development and deployment of effective vaccines, especially mRNA vaccines, and through the use of multidisciplinary technologies, the development of mRNA vaccines for cancer and infectious diseases has evolved^83^. mRNA vaccines are rapidly emerging as promising and impactful platforms for cancer treatment, providing targeted and efficient treatment alternatives that have demonstrated efficacy in preclinical investigations involving solid tumors^84,85^.

The onset of mRNA vaccine technology represents a transformative landmark in the development of tumor vaccines^86,87^. While conventional prophylactic vaccines against infectious diseases typically use attenuated viruses or inactivated pathogens as their antigen source, therapeutic mRNA vaccines for cancer take a radically different approach, offering key advantages: (1) eliminating safety concerns associated with live pathogens, and (2) enabling rapid customization to target patient-specific TSAs. Furthermore, when compared to other nucleic-acid-based delivery platforms like viral vectors, mRNA vaccines avoid the issue of preexisting immunity, which can dampen efficacy^88–91^.

The evolution of mRNA vaccines for PC thus exemplifies the effective implementation of basic scientific discoveries in clinical applications^92,93^.

### Molecular architecture of mRNA vaccines

**Fig. 3:**
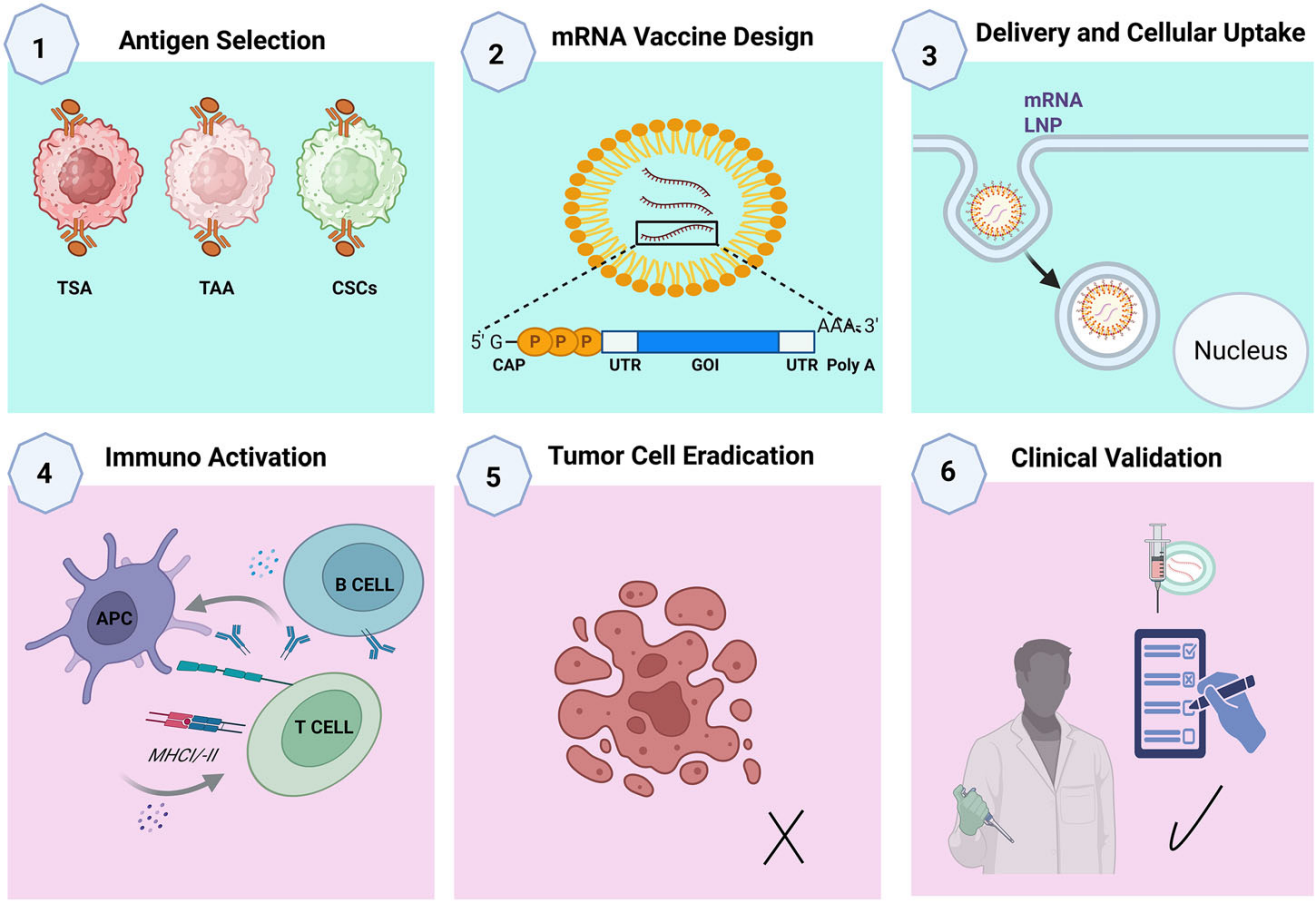
Mechanistic overview of mRNA-based vaccine development for PC: from antigen selection to clinical validation. This figure summarizes the sequential steps involved in the development and therapeutic action of mRNA vaccines for PC, highlighting key biological processes and immunological targets: Antigen selection: Tumor-associated antigens (TAAs) commonly overexpressed in PC such as prostate-specific antigen (PSA), prostatic acid phosphatase (PAP), and prostate-specific membrane antigen (PSMA) are identified as optimal targets due to their tumor-restricted expression and immunogenic potential. These antigens serve as the foundational components for vaccine design, aimed at eliciting specific T cell responses. mRNA vaccine design: codon-optimized mRNA sequences encoding selected TAAs are synthetically engineered to enhance translational efficiency, stability, and immunogenicity. Essential structural components include a 5′ cap, untranslated regions (UTRs), coding region, and a 3′ poly-A tail. Modified nucleosides (e.g., pseudouridine) are often incorporated to reduce innate immune recognition and prolong transcript half-life. Delivery and cellular uptake: mRNA molecules are encapsulated within lipid nanoparticles (LNPs) to protect them from degradation and facilitate targeted delivery. Upon intramuscular or intradermal administration, LNPs are preferentially taken up by professional antigen-presenting cells (APCs) such as dendritic cells via endocytosis, enabling intracellular mRNA release. Immunoactivation: Within APCs, the mRNA is translated into the encoded antigen, which undergoes processing and is presented on MHC class I and II molecules. This results in the activation of CD8⁺ cytotoxic T lymphocytes (CTLs) and CD4⁺ helper T cells, driving a robust and sustained adaptive immune response capable of recognizing and attacking tumor cells. Tumor cell eradication: activated CTLs infiltrate the TME and selectively destroy PC cells presenting the target antigens. CD4⁺ T cells support this response through cytokine production and enhancement of CTL function and memory formation. Clinical validation: promising preclinical findings advance to clinical trials, where safety, immunogenicity, and therapeutic efficacy of the mRNA vaccine are evaluated. Integration with ICIs or TME-modifying agents is under investigation to overcome immune suppression in advanced PC.

The effectiveness of the mRNA vaccine process, which is summarized in Fig. 3, largely hinges on maintaining the structural integrity and stability of the mRNA molecule^94–96^. The 5′ cap structure plays an essential role in protecting mRNAs from degradation and initiating translation, whereas the 3′ polyadenylated (poly-A) tail further stabilizes the transcript and enhances protein synthesis^97,98^. Additionally, the untranslated regions (UTRs) flanking the coding sequence play important roles in modulating the mRNA half-life and the efficiency of protein synthesis^99^. Chemical modifications such as the inclusion of pseudouridine or 5-methylcytidine have been employed to increase mRNA stability and reduce innate immune activation and are routinely employed to mitigate innate immune recognition and enhance translational output, thus optimizing the balance between immunogenicity and tolerability^100^. The molecular architecture of mRNA vaccines is meticulously engineered to maximize stability, translational efficiency, and immunogenicity.

The coding sequence itself is optimized to encode TSAs such as PSA, PSMA, and STEAP1 via codon optimization strategies to increase protein expression. A poly-A tail at the 3’ end further stabilizes the mRNA and facilitates translation^101^. Key modifications, including chemically modified nucleosides such as pseudouridine and 5-methylcytidine, can enhance mRNA performance, help evade innate immune sensors and enhance translational output. CureVac’s RNActive® platform exemplifies these innovations by integrating GC-rich sequences and protamine complexation to potentiate immune activation. To protect mRNAs from extracellular RNases and enhance intracellular delivery, LNPs are employed, while chemical modifications to the RNA backbone extend their intracellular persistence, collectively ensuring a robust and sustained antigen expression profile for effective vaccination^102^.

### Antigen selection in mRNA-based PC vaccines: integrating TSAs and TAAs

Effective mRNA vaccine development for PC hinges on strategic antigen selection, which involves the integration of both TAAs and TSAs to adequately address the profound inter- and intratumoral heterogeneity characteristics of PC^103^. Unlike tumors with more homogeneous antigenic profiles, PC comprises multiple subclonal populations, necessitating combinatorial or personalized vaccines approaches designed to generate robust and long-lasting immune responses^104–106^. Neoantigens derived from early/clonal mutations or from tumor-initiating/stem-like cell compartments represent an attractive vaccine target in PC because they are more likely to be shared across tumor clones and less prone to immune escape; preclinical studies and early-phase neoantigen vaccine trials support feasibility and immunogenicity of this strategy and justify further investigation in PC-specific trials (e.g., pilot neoantigen vaccine studies and PRAD antigen discovery effort)^107,108^.

Compared with healthy tissues, TAAs are frequently overexpressed in malignant prostate tissue and function as foundational targets in PC vaccine development. Notable examples include PSA, PAP, and PSMA, all of which have been leveraged in mRNA vaccines to initiate antigen-specific cytotoxic and helper T-cell responses. These antigens, when encoded into mRNA sequences encapsulated in LNPs, are delivered to professional APCs, such asDCs, where they are translated, processed, and presented via both the MHC class I and II pathways. This dual presentation enables the activation of CD8⁺ CTLs and CD4⁺ helper T cells, leading to a more robust and sustained antitumor immune response, which is summarized in Fig. 3^109–112^.

A key example is CV9103, a self-adjuvanting mRNA vaccine that encodes multiple TAAs, including PSA, PSCA, PSMA, and six-transmembrane epithelial antigen of the prostate 1 (STEAP1). These targets were selected on the basis of their overexpression or cancer-specific expression patterns in PC tissues versus benign prostate tissue, a feature that is essential for minimizing off-target toxicity and preventing autoimmunity. Additional TAAs, such as survivin, mucin-1 (MUC1), c-MYC, p53, and PTEN, have also been explored for inclusion in vaccine constructs because of their oncogenic roles and immunogenic potential, which is summarized in Table 3^113^.

Beyond TAAs, TSAs, particularly those derived from tumor-specific somatic mutations or gene fusions, offer greater tumor specificity and are central to the next generation of personalized mRNA cancer vaccines. TSAs are absent from healthy tissues and thus eliminate immune responses with a reduced risk of central tolerance or autoimmune reactivity^12^.

In PC, several neoantigen-derived TSAs, including SPOP mutations, FOXA1 mutations, and TMPRSS2-ERG gene fusions, have been identified through high-throughput bioinformatic and genomic analyzes. These aberrations result in the generation of novel peptide sequences uniquely expressed in tumor cells and presented via MHC molecules to T cells. Importantly, emerging bioinformatic studies have proposed a panel of promising TSA candidates on the basis of tumor specificity, high MHC binding affinity, and immunogenicity. These include Kelch-like protein 17 (KLHL17), carnitine palmitoyltransferase 1B (CPT1B), LCK-interacting transmembrane adaptor 1 (LIME1), IQ motif-containing GTPase-activating protein 3 (IQGAP3), YJEFN3, KIAA1529, cadherin EGF LAG seven-pass G-type receptor 3 (CELSR3), and MutS homolog 5 (MSH5)^114,115^. These molecules were selected for their high expression in malignant cells, absence or low levels in healthy tissues, and capacity to bind strongly to MHC molecules, criteria critical for effective T-cell recognition and activation. Furthermore, another study identified four additional candidates, FUS, RNPC3, LMNB2, and ZNF700, that were associated with worse prognosis and poor APC infiltration, suggesting that their expression may characterize immunologically “cold” tumors^116^. These targets may represent actionable TSAs for improving vaccine efficacy in patients with low baseline immune activity. Importantly, a correlation was observed between certain neoantigens and distinct immune phenotypes, particularly those with elevated B-cell infiltration, indicating that such patients may be optimal candidates for neoantigen-based vaccination strategies^117^. Most investigations so far have identified candidate neoepitopes using in silico prediction pipelines; however, the resulting neoantigens are generally patient-specific or confined to limited molecular subgroups rather than being universally shared across the wider PC population^118^. The effective implementation of mRNA-based cancer vaccines in PC depends not only on antigen selection but also on nuanced insight into the tumor immune microenvironment^119^.

In PC, Sipuleucel-T trials have shown that higher APC activation, total nucleated cell counts, and detectable antigen-specific responses correlate with longer OS^120^. In other cancers, such as melanoma and NSCLC, studies of personalized neoantigen vaccines (e.g., NEO-PV-01) have identified high tumor mutational burden, pre-existing TCR clonality, and presence of TLS or organized B-cell signatures as predictive biomarkers for response^121^. These findings suggest that patients whose tumors harbor a pre-existing but suppressed immune repertoire may represent the optimal phenotype for neoantigen vaccination in PC^122^.

Therefore, selecting appropriate TSAs/TAAs must be accompanied by immunophenotyping to identify and select patients who are most likely to respond favorably to vaccination^123,124^. In summary, comprehensive multiparameter strategies, including TSA/TAA identification, TME profiling, and personalized delivery, are fundamental for the rational design of potent mRNA vaccines for PC. Continued research integrating genomic, transcriptomic, and immunologic data will be necessary to refine target selection and maximize the therapeutic impact of these vaccines in both early-stage and advanced PC settings^125^.

### Delivery systems: engineering efficient mRNA uptake

The success of mRNA vaccines critically depends on efficient delivery to APCs, which orchestrate adaptive immune responses. Naked mRNA is inherently unstable and rapidly degraded by extracellular RNases, making protective delivery systems essential for stability, cellular uptake, and cytoplasmic translation. Current delivery modalities include LNPs, polymeric nanoparticles, polypeptide carriers, lipid–polymer hybrids, extracellular vesicles, viral vectors, and cell-based vehicles^94,126^.

Among these, LNPs are the most clinically advanced and widely used platform. They protect mRNA from enzymatic degradation, enhance cellular uptake via endocytosis, promote endosomal escape, and ensure cytoplasmic delivery for efficient antigen translation. Advances in lipid chemistry have further optimized LNP pharmacokinetics, biodistribution, and immunogenicity, enabling successful preclinical and clinical applications in cancer immunotherapy, including PC. Despite these advantages, LNPs have notable limitations. They can induce hepatotoxicity, infusion-related reactions, and display limited tissue selectivity, which may restrict therapeutic efficacy in solid tumors with dense stroma and immunosuppressive microenvironments. To address these challenges, alternative and emerging platforms are under investigation. Polymeric nanoparticles offer tunable stability and controlled release, lipid–polymer hybrids combine the advantages of lipids and polymers for improved uptake and safety, extracellular vesicles provide natural tissue targeting with reduced immunogenicity, and peptide-based carriers enhance cellular penetration and tumor specificity. These approaches hold promise for improving tumor-specific delivery, reducing systemic toxicity, and overcoming the barriers of the prostate cancer microenvironment^127,128^.

Next-generation delivery strategies for neoantigen vaccines include lipid–polymer hybrid nanoparticles (LPPs) and chemically synthesized minimal mRNA (CmRNA). CmRNA, lacking UTRs and polyadenylation, shows enhanced stability and translational efficiency, while LPP encapsulation improves uptake, target specificity, and safety compared to traditional LNPs. These platforms enable efficient CD8+ T-cell activation and personalized cancer immunotherapy, offering a promising alternative to conventional IVT-mRNA and circular RNA approaches, though further optimization and clinical evaluation are needed^129^.

An ideal mRNA delivery system must satisfy four critical criteria: (1) protect the mRNA cargo from degradation, (2) enable efficient uptake into target cells, (3) permit controlled release and cytosolic delivery, and (4) minimize nonspecific immune activation. LNPs excel across all these parameters and are now considered the gold standard for both prophylactic and therapeutic mRNA vaccine formulations^130–133^.

### Mechanism of immune activation: from mRNA translation to tumor cell elimination

Upon LNP-mediated delivery, the mRNA is released into the cytoplasm, where ribosomes translate it into the encoded tumor-specific proteins. This intracellular synthesis mimics natural antigen production and allows APCs, particularly DCs, to process and present antigens via both MHC class I/II pathways. The antigenic peptides are either degraded by the proteasome and loaded onto MHC I for CD8⁺ CTL activation or trafficked to lysosomes for degradation and presentation on MHC II to activate CD4⁺ helper T cells^90,134^.

This dual-presentation pathway is important for the induction of a comprehensive and prolonged antitumor immune response involving both the cellular (CD8⁺ and CD4⁺ T cells) and humoral (B cells) arms of the immune system. Importantly, mRNA vaccines can encode full-length tumor antigens or multiple neoantigens, enabling broad epitope coverage and robust T-cell priming independent of the HLA haplotype. This contrasts with peptide- or DNA-based vaccines, which often suffer from limited immunogenicity and restricted HLA compatibility^135^.

Furthermore, the inclusion of immunostimulatory adjuvants within mRNA constructs, such as unmethylated CpG motifs or activation domains for innate immune receptors (e.g., TLRs and RIG-I), enhances DC maturation, cytokine secretion, and the polarization of T cells toward a Th1-type response. This ensures that a proinflammatory TME is conducive to immune-mediated tumor clearance^136^.

Conventional PC therapies, including radioligand therapy, benefits only 20–30% of PC patients because of the intratumoral heterogeneity in PSMA/PSCA expression and therapeutic resistance observed in the trial^113^. Similarly, ADCs such as MLN2704 (a PSMA-targeting agent conjugated to a cytotoxic payload) and bispecific antibodies targeting PSMA and STEAP1 are limited by systemic toxicity and short serum half-lives^137–141^.

In contrast, mRNA vaccines offer several unique advantages. Unlike many peptide- or viral-vector-based cancer vaccines that typically deliver only a limited number of defined epitopes, mRNA vaccines can encode full-length antigens or multiple antigens simultaneously. This broadens the repertoire of epitopes presented across different HLA types, reducing the risk of immune escape due to the loss or downregulation of a single antigenic epitope. Moreover, because mRNA vaccines are not based on viral vectors, they avoid the problem of pre-existing or rapidly induced anti-vector immunity, which can compromise booster dosing and limit the number of antigens that can be delivered. The mRNA platform’s rapid adaptability also enables the creation of personalized vaccines tailored to the unique mutational landscape of a patient’s tumor^142^.

We clarify that while both modalities are ultimately hindered by the immunosuppressive TME during the effector phase, vaccines possess a key advantage: the ability to generate a de novo T-cell response. This allows them to bypass the fundamental limitation of ICIs in PC, which lack the pre-existing T-cell infiltrate necessary for ICI activity. Consequently, vaccines can potentially “heat up” a cold tumor, creating a population of tumor-specific T cells that could then be further amplified by ICIs in a combination approach^54^.

However, we also detail the distinct challenges unique to vaccines. Their success is critically dependent on optimal antigen selection, as tumor heterogeneity or antigen loss can render the vaccine-primed T cells ineffective. Furthermore, generating a robust and durable T-cell response requires overcoming immune tolerance to self-antigens, a hurdle not faced by ICIs. In contrast, ICIs contend with a different set of challenges, primarily their propensity for broad, systemic immune activation, which leads to a higher risk of immune-related adverse events compared to the more targeted action of vaccines. This comparative analysis underscores that while vaccines can circumvent the problem of pre-existing immunity, they introduce unique complexities related to antigen choice and the quality of the induced immune response^55^.

### Optimization of mRNA constructs for PC immunotherapy

To increase mRNA stability, translational efficiency, and immunogenicity, several structural optimizations have been employed. These include the inclusion of modified nucleosides, such as pseudouridine-optimized 5′ caps and poly(A) tails, GC-enriched coding sequences, and tailored UTRs^143^.

The use of high-throughput transcriptomic and proteomic analyzes also enables antigen selection by identifying genes with optimal expression levels, proteasomal processing characteristics, and strong MHC binding affinity. This computational pipeline ensures that selected TSAs are both immunogenic and tumor-specific^144^.

### Clinical considerations and immunological safety

Despite their advantages, mRNA vaccines may trigger immune phenomena such as pseudoprogression, where immune cell infiltration mimics tumor growth on imaging, can complicate the clinical assessment of therapeutic efficacy, particularly in immunologically active tumors^145,146^. While this may indicate effective immune activation, it can complicate radiological assessment and must be distinguished from true disease progression. Careful monitoring of immune-related adverse events is therefore essential in clinical settings^147^.

The transient expression kinetics of mRNAs reduce the risk of chronic inflammation and autoimmunity while allowing for the induction of immunological memory. Compared with CAR-T-cell therapy, which is limited by limited tumor access and antigen specificity, mRNA vaccines offer a more flexible and safer platform for systemic antitumor immunity^148^.

### Advantages vs. disadvantages of mRNA vaccines

Traditional cancer immunotherapies often struggle against immunologically “cold” tumors, and approaches such as checkpoint inhibitors or CAR-T cells are limited by the immunosuppressive TME. In contrast, mRNA-based platforms offer a flexible means of delivering not only TSAs but also modulators of the TME in the same formulation. For example, mRNA can encode cytokines such as IL-12 or IL-15 to stimulate T-cell and NK-cell activity; co-stimulatory ligands such as OX40L or 4-1BBL to enhance T-cell activation; or receptors such as a dominant-negative TGF-β receptor to block immunosuppressive signals. This dual delivery strategy enables both precise immune priming and localized immunomodulation within the tumor site, potentially overcoming key barriers to effective cancer immunotherapy^149^. mRNA vaccines can encode longer antigens or full-length proteins, allowing for endogenous processing and presentation of a broader repertoire of epitopes across different HLA alleles and thus covering a more diverse patient population. mRNA vaccines represent an innovative approach with distinct benefits and limitations in PC treatment. Their most compelling advantage lies in their rapid development capabilities, enabling swift adaptation to emerging tumor antigens or resistance mutations. This flexibility is complemented by their noninfectious nature and inability to integrate into host DNA, eliminating the risks of infection or insertional mutagenesis observed with viral vectors. Unlike traditional peptide vaccines, mRNA platforms can stimulate both antibody production and T-cell responses an important advantage for comprehensive tumor elimination^150,151^.

mRNA vaccines are a promising strategy for PC, particularly after surgery to eliminate remaining microscopic disease. The cancer’s slow growth allows time for the immune system to be trained, and the vaccine’s natural design avoids long-term inflammation. However, significant challenges remain. The mRNA is fragile and requires complex delivery systems such as LNPs, and efficient targeting of APCs remains technically demanding. It can be difficult to target the right immune cells, and the strong immune response can sometimes cause harmful inflammation or be mistaken for cancer growth on scans. Furthermore, these personalized vaccines are expensive to make, difficult to store, and their long-term effectiveness and best use in treatment plans are still under investigation^152–154^.

### Future of mRNA vaccines

The future of mRNA vaccines in PC will depend on overcoming these delivery and manufacturing challenges while demonstrating durable clinical efficacy. Current trials exploring combination approaches with existing therapies may help establish their role in the PC treatment paradigm. Their unique ability to rapidly adapt to tumor evolution while stimulating comprehensive immune responses positions them as a potentially transformative approach, provided that these technical and clinical hurdles can be adequately addressed, as summarized in Scheme 2.

A further advantage lies in the presence of PSA as a well-established biomarker, enabling precise monitoring of the disease course and treatment efficacy. Moreover, mRNA vaccines possess inherent immunostimulatory properties by engaging pattern recognition receptors, such as TLR3, TLR7, and TLR8, thereby increasing immune activation without requiring external adjuvants. The transient expression profile of mRNA-encoded antigens also minimizes the risk of chronic inflammation and autoimmunity^155,156^.

Nevertheless, mRNA vaccine platforms are not without limitations. The inherent instability of mRNA necessitates encapsulation in protective carriers, such as LNPs, to facilitate delivery and ensure efficient translation within target cells^157^. While innate immune activation supports adjuvanticity, excessive stimulation may hinder antigen expression and provoke undesirable systemic effects^158^. Furthermore, variability in antigen processing and presentation can limit the consistency and breadth of immune responses across individuals^159^.

### Preclinical development pathway of PC vaccines

The preclinical development of PC vaccines has advanced considerably over time. In 1996, researchers isolated autologous DCs from PC patients and cultured them ex vivo with either autologous tumor cell lysates or HLA-A2-restricted peptide epitopes derived from PSMA^160^. These DCs were subsequently employed to stimulate CTLs in vitro. With this approach, researchers later transfected autologous DCs with mRNA encoding PSA, which, when administered to PC patients, elicited rapid and measurable T-cell responses, marking an important step forward in personalized immunotherapy strategies^161^.

Viral and bacterial vector-based vaccines, including the orthopoxvirus or vaccinia virus in the PROSTVAC-V vaccine and fowl pox in the PROSTVAC-F vaccine, as well as *Listeria monocytogenes* in the ADXS31-142 vaccine, have been used to deliver PC-related antigens^162–164^. These vaccines operate on the principle that cells will naturally recognize these vectors, allowing their nucleic acid to replicate within the cytoplasm and trigger immune responses^162,165^. While the results have been mixed, most studies have demonstrated the feasibility and safety of these vaccines, which are frequently accompanied by indications of prolonged survival and enhanced immune activation, underscoring their potential as viable therapeutic options in PC^1^. However, immune responses to the vector itself and diminished T-cell responses in some cases have been observed. Nucleic acid vaccines, particularly mRNA-based vaccines, offer several advantages over virus- and bacteria-based vaccines by encoding only TSAs, which avoids genome integration and reduces immune reactions against vectors, thus increasing safety and stability^166,167^.

DNA plasmid vaccines, such as those encoding PSA, PSMA, the ligand-binding domain of the androgen receptor (pTVG-AR), and (PAP, pTVG-HP), have also been explored in clinical trials^26^. These vaccines can stimulate T-cell responses, reduce PSA levels, and demonstrate efficacy when combined with ICIs^168,169^. In preclinical models of PC, mRNA vaccines have shown promising results, including 50–70% tumor regression, a threefold increase in TILs, a 30% survival benefit in metastatic models, and a significant reduction in the incidence of bone metastasis^170,171^. These outcomes highlight the dual ability of mRNA vaccines to both induce cytotoxic T-cell responses and reprogram the immunosuppressive TME^172^.

### Clinical translation: from lab bench to patient bedside or clinical trials II/III

Sipuleucel-T remains the only FDA-approved therapeutic cancer vaccine for PC proven to prolong OS. Its success is attributed not only to inducing antigen-specific T-cells but also to its ability to modulate the immunosuppressive TME^173^. In contrast, other vaccine platforms have faced significant challenges in late-stage development. For instance, GVAX® (Cell Genesys, Inc.), a GM-CSF-secreting whole-cell vaccine, yielded conflicting efficacy data across clinical trials. While some early-phase studies suggested potential survival benefits, particularly when combined with immune checkpoint inhibitors (ICIs) or androgen deprivation therapy (ADT), a pivotal Phase 3 trial was halted due to futility and an observed increase in the rate of death compared to the control arm^174^. Similarly, the autologous DC vaccine DCVAC/PC failed to demonstrate a significant improvement in OS in its Phase 3 trial, likely due to variable antigen presentation and insufficient immunogenicity, leading to its discontinuation^175^. These outcomes underscore the hurdles in achieving consistent and robust efficacy with cancer vaccines.

Among newer approaches, mRNA-based vaccines have emerged as a promising strategy. In phase I/II trials, CV9103 showed immune activation with acceptable safety but limited clinical benefit (PSA progression-free survival 15.9% at 6 months; median survival 31.4 months in metastatic patients)^176^. Its successor, CV9104, induced T-cell responses in 64% of patients in a larger phase IIb trial (*n* = 197) but did not improve OS, underscoring the need to optimize mRNA vaccine strategies for clinical efficacy^177^.

Despite their favorable safety profiles, mRNA vaccines have yet to substantially improve radiographic progression, PSA levels, or long-term survival in patients with PC. Challenges include optimizing antigen selection, enhancing mRNA stability, and overcoming the immunosuppressive TME^178,179^. Advances in LNP delivery systems and multiantigen vaccines (e.g., the Tetra vaccine) show preclinical promise by enhancing lymph node targeting and T-cell activation^180^. However, clinical translation remains hindered by the need for better patient stratification, combination strategies with ICIs or ADT, and long-term safety monitoring for potential autoimmune effects^181^.

Moreover, other investigational vaccines, including DC-based therapies (NCT01446731, NCT01278914), peptide vaccines (NCT02452307), and RNActive® vaccines (NCT02140138), continue to explore diverse antigen targets and delivery platforms. Early-phase trials, such as those testing MUC-2-KLH conjugate vaccines (NCT00698711), highlight ongoing efforts to refine immunotherapeutic approaches. While progress has been incremental, these studies underscore the need for deeper mechanistic insights and optimized vaccine designs to improve clinical outcomes in PC^26,182,183^, as outlined in Table 4.

**Table 4:** Summarized clinical trial status and outcome

Vaccine name	Antigens targeted	Clinical trial phase	Year initiated	Key findings	Source
CV9103	PSA, PSMA, PSCA, STEAP1	I/II	2016	Demonstrated safety and induced immune responses in patients with advanced PC.	^214^
CV9104	Six PC-associated antigens	I/II	2016	Showed promising immunogenicity and is under evaluation in castration-resistant PC (CRPC) and high-risk settings.	^215,216^
DC vaccine with docetaxel	PSA, PAP, survivin and hTERT	II	2011	Dendritic cell vaccination in combination with docetaxel for patients with cancer prostate.	NCT01446731
Dendritic cells	PSA	I/II	2002	Determination of safety and toxicity of vaccination with mRNA transfected DC Determine immunological response to the vaccine.	NCT01278914^217^
Human telomerase reverse transcriptase messenger RNA (hTERT mRNA)	PSA	I/II	2008	Select the arm with the highest biologic response and the first ranked arm will be selected for further study in a larger efficacy trial.	NCT01153113^106^
Dendritic cell vaccine	PSA	I/II	2010	Patients receiving DC-vaccination may have a reduced risk of PSA relapse or increased time to PSA relapse.	NCT01197625
Peptide vaccine	PSA	I/II	2004	evaluates the PSA response in HLA-A*02 positive patients treated with a prostate-specific peptide vaccine in combination with different immune-adjuvants.	NCT02452307
RNActive® vaccine	PCA	II	2014	Evaluate the induction of immune responses against CV9104 administered by conventional intradermal injection or with a needle-free intradermal injection device.	NCT02140138
mDC and pDC vaccination	Human leukocyte antigen, PSA	II	2015	Show immunologic efficacy of tumor-peptide loaded natural DC in metastatic castration-resistant prostate cancer patients (mCRPC).	NCT02692976^218^
MUC-2-KLH	PSA	I	1997	Vaccination of Prostate Cancer Patients With MUC-2-KLH Conjugate Plus the Immunological Adjuvant QS21.	NCT00698711

### The cutting edge: next-generation innovations

AI is rapidly becoming a transformative force in the development of mRNA vaccines for PC, with its success largely driven by innovations in machine learning (ML) and deep learning (DL) technologies. These tools enable precise prediction of neoantigens, optimization of mRNA sequences, and personalized vaccine design by analyzing vast genomic and immunological datasets, thereby accelerating the transition from bench to bedside^184^. These technologies can analyze vast and complex datasets spanning genomic, proteomic, and clinical information to identify TSAs with a high degree of precision^185^.

ML models, including DL architectures including recurrent neural networks (RNNs) and convolutional neural networks (CNNs), play pivotal roles in the development of mRNA vaccines. CNNs are particularly effective in analyzing spatial patterns within biological data, such as tissue images or sequence motifs, whereas RNNs excel in processing sequential data such as RNA or protein sequences. These models aid in identifying optimal antigenic targets, predicting RNA secondary structures, and enhancing mRNA stability and translational efficiency, which are key factors in the design of effective PC vaccines, and excel at uncovering subtle patterns and relationships in data^186^. This capability is critical for pinpointing the most promising TAAs that can be targeted by mRNA vaccines to elicit a robust immune response^126^.

Owing to its hierarchical network structure and advanced feature extraction capabilities, DL plays an important role in refining the design of mRNA vaccines^187^. By leveraging predictive algorithms, DL models can optimize the mRNA sequence, selecting the most effective codon usage and necessary nucleotide modifications to increase vaccine stability, translation efficiency, and immunogenicity^188^. This computational approach not only streamlines the vaccine design process but also substantially enhances the precision and therapeutic efficacy of the resulting vaccine constructs^189,190^.

In the realm of personalized medicine, both ML and DL are indispensable. PC exhibits significant heterogeneity among patients, necessitating personalized therapeutic approaches^191^. Advanced algorithms can integrate patient-specific molecular profiles to identify unique mutations and antigenic variations, thereby enabling the development of tailored mRNA vaccines^192^. This personalized strategy ensures that each vaccine is customized to target the distinct molecular drivers of an individual’s cancer, potentially enhancing treatment efficacy while minimizing adverse effects^187,193^.

In addition to vaccine design, these technologies are instrumental in optimizing vaccine delivery systems^194,195^. ML and DL algorithms can simulate and refine LNP formulations to guarantee the efficient delivery of mRNAs to target cells for optimal therapeutic outcomes and maintain their structural integrity in vivo^196,197^. Furthermore, these algorithms assist in predicting patient responses to a vaccine, which is invaluable for designing adaptive clinical trials. By stratifying patients on the basis of predictive biomarkers, clinical trials can be more efficiently structured, focusing on those most likely to benefit and thus accelerating both development timelines and regulatory approvals^198^.

Finally, real-time monitoring of immune responses is significantly enhanced through the application of ML and DL^196^. These models analyze longitudinal data from biomarker fluctuations to immune cell dynamics to detect early indicators of treatment resistance or adverse effects. Such insights enable clinicians to adjust treatment regimens promptly, ensuring both the safety and efficacy of the therapy over time^71,93,199^.

**Fig. 4:**
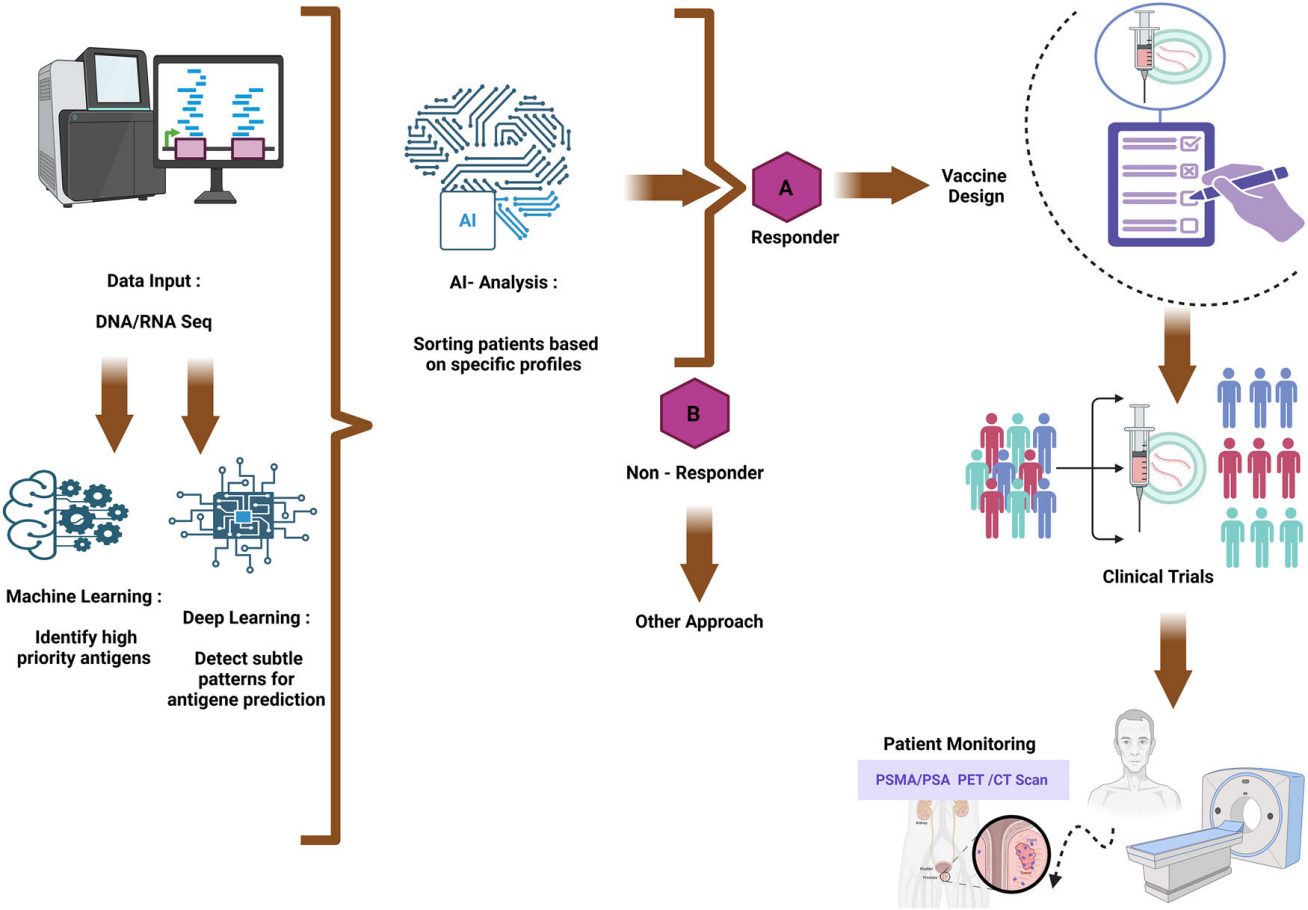
Role of AI in the design and development of mRNA vaccines for PC. This figure illustrates the integration of artificial intelligence (AI) encompassing machine learning (ML) and deep learning (DL) across multiple stages of mRNA vaccine development for PC. These technologies are transforming vaccine research into a more precise, efficient, and personalized process. Antigen prediction and neoantigen prioritization: AI algorithms analyze genomic, transcriptomic, and proteomic datasets to predict tumor-specific antigens and neoantigens with high immunogenic potential. ML models assess peptide-MHC binding affinities, antigen processing likelihood, and immune escape risks, enabling the selection of optimal mRNA vaccine targets. mRNA sequence optimization: DL models aid in designing synthetic mRNA constructs with improved stability, efficient codon usage, and enhanced translational performance. These tools optimize key elements such as the 5′ cap, untranslated regions (UTRs), and poly-A tails to increase protein expression and reduce immunogenic side effects. Nanoparticle delivery system design: AI-driven modeling supports the rational design of lipid nanoparticles (LNPs) with ideal physicochemical properties for efficient mRNA encapsulation, protection, and cellular delivery. Predictive algorithms optimize lipid composition, charge, and particle size to enhance APC uptake and endosomal escape. Immune Response prediction and simulation: computational models forecast vaccine-induced immune responses, including T cell activation profiles and cytokine dynamics. These predictions help guide preclinical testing and prioritize candidate formulations for clinical development. Personalized vaccine development: AI integrates patient-specific tumor profiles to design individualized vaccine constructs, enabling precision immunotherapy tailored to each patient’s mutational landscape and immune contexture. Treatment monitoring: Real-time analysis of clinical and molecular data using AI allows dynamic assessment of vaccine efficacy, immune biomarkers, and tumor evolution, facilitating adaptive treatment strategies and early intervention.

In summary, the integration of ML and DL into the development of mRNA vaccines for PC represents a significant leap forward in precision oncology. These advanced computational techniques facilitate more targeted vaccine design, efficient delivery, and continuous treatment monitoring, paving the way for personalized and highly successful cancer immunotherapies. As these technologies advance, they hold great potential to refine and accelerate the development of next-generation cancer therapies, ultimately providing new hope in the battle against PC, which is summarized in Fig. 4^200^.

However, the clinical application of mRNA vaccines in PC faces several challenges. Key limitations include the identification of suitable TSAs, the selection of optimal vaccination candidates, and the paucity of large-scale clinical trials to validate their efficacy and safety^117^. Additionally, the complex interplay between the TME and the immune system further complicates their implementation. Despite these challenges, the transformative potential of mRNA vaccines to redefine PC treatment remains substantial. Future research should address these limitations through the development of predictive biomarkers, innovative delivery systems, and combination therapies to improve therapeutic outcomes. By overcoming these obstacles, mRNA vaccines have the potential to usher in a new era of precision medicine in PC, providing renewed hope for increased survival rates and improved quality of life for patients.

### Road ahead, challenges and opportunities in mRNA vaccines

Complementing these clinical efforts, AI-driven antigen discovery has identified next-generation targets (KLHL17, carnitine palmitoyl transferase 1B (CPT1B), etc.) that address the antigen selection challenge noted earlier. These computationally selected antigens show 3x greater tumor specificity than traditional targets in preclinical validation, enabling more potent vaccine designs while maintaining the favorable safety profile of mRNA platforms^187^.

The numerous advantages of cancer vaccines in treating cancer, which are equally important, are the recognition and management of their inherent limitations and challenges. Key obstacles to the efficacy of cancer vaccines include potential side effects, tumor heterogeneity, the immunosuppressive nature of the TME, and mechanisms of immune tolerance. However, mRNA vaccines have several advantages over other tumor vaccines, such as increased specificity, convenient synthesis, time and cost benefits, safety, and a low risk of genomic mutation^201^. The benefits and drawbacks of mRNA vaccines are contingent upon the specific type of mRNA vaccine utilized. For example, mRNA-loaded DCs may offer enhanced efficiency and tolerability; however, they entail higher costs associated with cell culture and intricate pharmacokinetics. In contrast, direct injection of mRNA vaccines is a more economical and straightforward administration method; however, it is characterized by a shorter half-life and reduced immunogenicity^202^.

While mRNA-based therapeutic vaccines remain in the developmental phase for PC, their distinct advantages, including rapid manufacturability and multiantigen targeting, position them as a transformative immunotherapeutic approach, particularly for advanced disease. Future advancements hinge on three key pillars: the discovery of novel TSAs through AI-driven genomics, stratification of patients by immune-responsive subtypes, and rational combination strategies with ICIs or adoptive cell therapies to overcome the immunosuppressive TME. These directions align with the broader paradigm shift toward precision immunotherapy in PC management^106,203^.

### Final vision: integrating mRNA vaccines into standard oncology practice

The journey of mRNA vaccines from a promising concept to PC treatment is underway. The ultimate goal of this review is not to replace existing therapies, but to weave mRNA vaccines into the standard oncological toolkit, creating a new, more powerful paradigm for patient care. The path to this future is defined by three critical, interconnected fronts: A. Smarter Delivery: The next generation of vaccines will move beyond basic LNPs. We will see platforms engineered for precise targeting of lymph nodes and even self-amplifying mRNA (saRNA) designs that create a stronger, longer-lasting immune response with a smaller dose. The vehicle for the vaccine will become as sophisticated as its genetic code^204^. B. True Personalization: The era of “one-size-fits-all” cancer vaccines is ending. The future lies in using AI and rapid sequencing to create bespoke vaccines tailored to the unique neoantigens of an individual’s tumor. This is the key to overcoming tumor heterogeneity and preventing immune escape^205^. C. Powerful Combinations: mRNA vaccines will rarely be used alone. Their greatest power will be unlocked by strategically combining them with other agents—such as ICIs or drugs that break down the tumor’s defensive shield—to create a synergistic attack on the cancer.

To bring this vision to life, the field must now focus on bridging the gap between promising science and clinical reality. This requires a concerted effort to: identify the Right Patients: Develop reliable biomarkers to predict who will benefit most. Prove Efficacy in Definitive Trials: Conduct large-scale Phase III trials that measure meaningful patient outcomes. Ensure Global Access: Streamline manufacturing to make these complex therapies scalable and affordable.

In conclusion, the message is one of optimistic determination. mRNA vaccine technology offers a fundamentally new way to combat prostate cancer one that is dynamic, personalized, and integrated. By focusing on these strategic priorities, we can transition these vaccines from experimental agents to cornerstone therapies. The collective effort of researchers, clinicians, and industry holds the power to not just treat prostate cancer more effectively, but to fundamentally redefine the patient journey, transforming a life-threatening disease into a manageable condition.
